# Maternal iodine status in a multi-ethnic UK birth cohort: associations with autism spectrum disorder

**DOI:** 10.1186/s12887-020-02440-y

**Published:** 2020-12-05

**Authors:** Kirsten Jade Cromie, Diane Erin Threapleton, Charles Jonathan Peter Snart, Elizabeth Taylor, Dan Mason, Barry Wright, Brian Kelly, Stephen Reid, Rafaq Azad, Claire Keeble, Amanda H. Waterman, Sarah Meadows, Amanda McKillion, Nisreen A. Alwan, Janet Elizabeth Cade, Nigel A. B. Simpson, Paul M. Stewart, Michael Zimmermann, John Wright, Dagmar Waiblinger, Mark Mon-Williams, Laura J. Hardie, Darren Charles Greenwood

**Affiliations:** 1 Leeds Institute of Cardiovascular & Metabolic Medicine, School of Medicine, University of Leeds, Leeds, LS2 9JT UK; 2 Bradford Institute for Health Research, Bradford Teaching Hospitals NHS Foundation Trust, Bradford, BD9 6RJ UK; 3 The Hull York Medical School, University of York, Heslington, York, YO10 5DD UK; 4 Earth Surface Science Institute, School of Earth and Environment, University of Leeds, Leeds, LS2 9JT UK; 5 Leeds Institute for Data Analytics, University of Leeds, Leeds, LS2 9JT UK; 6 School of Psychology, University of Leeds, Leeds, LS2 9JT UK; 7 Elsie Widdowson Laboratory, Cambridge, CB1 9NL, UK NIHR Nutritional Biomarker Laboratory, MRC Epidemiology Unit, University of Cambridge, Clifford Allbutt Building, Hills Road, Cambridge, CB2 0AH UK; 8 School of Primary Care and Population Sciences, Faculty of Medicine, University of Southampton, Southampton General Hospital, Southampton, SO16 6YD UK; 9 NIHR Southampton Biomedical Research Centre, University of Southampton and University Hospital Southampton NHS Foundation Trust, Southampton, UK; 10 Nutritional Epidemiology Group, School of Food Science & Nutrition, University of Leeds, Leeds, LS2 9JT UK; 11 Division of Women’s and Children’s Health, School of Medicine, University of Leeds, Leeds, LS2 9JT UK; 12 Faculty of Medicine and Health, University of Leeds, Leeds, LS2 9JT UK; 13 Laboratory for Human Nutrition, Institute of Food, Nutrition and Health, ETH Zurich, 8092 Zürich, Switzerland; 14 Leeds Institute for Data Analytics, University of Leeds, Leeds, UK

**Keywords:** Autism spectrum disorder, Iodine, Deficiency, Fetal development, Thyroid, Pregnancy

## Abstract

**Background:**

Maternal iodine requirements increase during pregnancy to supply thyroid hormones essential for fetal brain development. Maternal iodine deficiency can lead to hypothyroxinemia, a reduced fetal supply of thyroid hormones which, in the first trimester, has been linked to an increased risk of autism spectrum disorder (ASD) in the child. No study to date has explored the direct link between maternal iodine deficiency and diagnosis of ASD in offspring.

**Methods:**

Urinary iodine concentrations (UIC) and iodine/creatinine ratios (I:Cr) were measured in 6955 mothers at 26–28 weeks gestation participating in the Born in Bradford (BiB) cohort. Maternal iodine status was examined in relation to the probability of a Read (CTV3) code for autism being present in a child’s primary care records through a series of logistic regression models with restricted cubic splines.

**Results:**

Median (inter-quartile range) UIC was 76 μg/L (46, 120) and I:Cr was 83 μg/g (59, 121) indicating a deficient population according to WHO guidelines. Ninety two children (1·3%) in our cohort had received a diagnosis of ASD by the census date. Overall, there was no evidence to support an association between I:Cr or UIC and ASD risk in children aged 8–12 years (*p* = 0·3).

**Conclusions:**

There was no evidence of an increased clinical ASD risk in children born to mothers with mild-to-moderate iodine deficiency at 26 weeks gestation. Alternative functional biomarkers of exposure and a wider range of conditions may provide further insight.

## Background

Iodine is a trace mineral present in certain foods. As an essential constituent for the synthesis of thyroid hormones, iodine plays a vital role in supporting the optimal functioning of metabolic processes at all stages of life [1]. Thyroid hormones (TH) are central to many of the fundamental processes which underlie the development and maturation of the pre-natal brain; namely facilitating neural migration and differentiation in the cerebral cortex and hippocampus [2, 3]. The maximal vulnerability to an imbalance of TH exists before the onset of thyroid function in the fetus, which occurs around 16–20 weeks of gestation [4]. During this time, the highly orchestrated chain of neurodevelopmental events which characterize the early fetal period are exclusively dependent on maternal TH supply.

A woman’s iodine requirements are increased by over 50% during pregnancy [5] in order to support normal neurological development, and to compensate for pregnancy-associated increases in renal iodine clearance [5]. Severe maternal iodine deficiency can lead to a reduced fetal TH supply [5] (*hypothyroxinemia)* which, during critical developmental periods, is thought to impair neuronal migration and has been linked to long-term suboptimal child development [1].

The importance of thyroid function for neurodevelopment has been widely recognised for over a century [6], this is reflected in initiatives such as the World Health Organization (WHO)‘s salt iodization programme, which is now standard practice in many parts of the world [7]. The widespread implementation of such interventions, in conjunction with improved general nutrition, has given rise to a diminishing prevalence of iodine deficiency disorders in Western Europe [1]. Nevertheless, iodine intake remains potentially inadequate, especially in countries such as the UK which have not implemented iodization programmes.

Suboptimal gestational iodine intake has been linked with an increase in Attention Deficit, Hyperactivity Disorder (ADHD) symptom scores in children [8], poorer child language and reading skills [9], as well as a heightened likelihood of the child receiving special education services at age 8 years [9]. Yet few studies have directly investigated the possible linkage between gestational iodine deficiency and developmental disorders, such as autism spectrum disorder (ASD) – a developmental disorder characterised by difficulties with social interaction and communication, and by restricted and repetitive patterns of behaviour [10].

Despite comprehensive genetic studies, the aetiology of ASD remains largely unknown [11] with a potentially important role of environmental factors during fetal development [12], possibly through interference with neuronal migration [13]. Severe maternal iodine deficiency causing transient gestational hypothyroxinemia in rodents has been shown to induce cortical neuronal migration brain lesions resembling those-characteristic of autism [14]. Findings from epidemiologic studies [15, 16] provide observational evidence to support the notion that early intrauterine exposure to insufficient thyroid hormone levels could be an important cause of ASD. Severe maternal hypothyroxinemia measured during the first trimester of pregnancy was associated with an increased risk of ASD identified through record linkage in the United States [15] and abnormal maternal thyroid functioning in early pregnancy was associated with ASD in the offspring in The Danish National Birth Cohort Case-Control Study [16]. However, a large cohort pooling project has found no associations with maternal iodine status across early or mid-pregnancy and autistic traits in children [17].

The uncertainties surrounding the potential implications of low maternal iodine status for the development of ASD are of heightened relevance given that insufficient iodine status has been reported amongst women of childbearing age and pregnant women in the UK [18, 19] The WHO guidelines from which these reports are based identify pregnant populations as having insufficient iodine intake where the median urinary iodine concentration (UIC) is less than 150 μg/L. [20] This threshold however, is based on theoretical assumptions about metabolic needs, absorption, and excretion [1], as opposed to observed outcomes and therefore is limited in its application to pregnant populations. Using linked datasets, the current study aims to directly address and explore these issues by investigating the association between maternal iodine status in mid-pregnancy and an ASD diagnosis in the child, across a continuous range of urinary iodine concentrations.

## Methods

### Study design and participants

This research uses data from the Born in Bradford (BiB) cohort, which consists of 12,453 women recruited at 26–28 weeks gestation from 2007 to 2010. All babies born to participants from the 13,776 pregnancies which occurred during this time were eligible for inclusion, there were no exclusion criteria for this sample. Iodine status was estimated from spot urine samples collected from 6644 women (53%) across 6996 pregnancies (some women participated during successive pregnancies). A detailed description of the BiB cohort can be found elsewhere [21]. In brief, the cohort reflects Bradford’s multi-ethnic population; with 43% of those with urine samples being of White British heritage and 43% being of Pakistani heritage. The cohort consists of over half of all children born at Bradford Royal Infirmary between 2007 and 2011 and is considered broadly representative of the wider population [21]. Ethical approval for BiB was granted by Bradford Research Ethics Committee (Ref 07/H1302/112). All participants provided written informed consent prior to data collection.

### Assessment of urinary iodine and creatinine

Urine samples were provided by participants at routine antenatal clinics for oral glucose tolerance tests following an overnight fast. All available samples were centrifuged, divided into aliquots and barcode tagged before being frozen and stored at − 80 °C at the Bradford Royal Infirmary until their transfer to the Leeds Institute of Cardiovascular and Metabolic Medicine, University of Leeds, UK for iodine and creatinine analysis.

Urinary iodine^(127)^ concentration (μg/L) was measured using inductively coupled plasma-mass spectrometry (Thermo iCAP Q, Liverpool, UK). The laboratory is accredited under the US Centers for Disease Control and Prevention (CDC) Ensuring the Quality of Urinary Iodine Procedures (EQUIP) standardisation programme [22]. A standard Jaffe reaction-based microplate assay was used for the evaluation of creatinine concentrations. A series of five quality control (QC) urines (along with a certified reference material (CRM) (Seronorm trace elements urine L-1)) were repeatedly analysed in conjunction with participant samples in order to validate method accuracy. All QC and CRM categories remained within expected or certified ranges and displayed low variability.

For inter-laboratory comparison, a subset of BiB and validation samples (3·8%, 271 of 6971), were provided at regular intervals to the MRC Elsie Widdowson Laboratory in Cambridge. UIC measurements were highly correlated across sites (r = 0·99). Details of the full laboratory analysis and validation protocols are provided in the supplementary material.

### Diagnosis of ASD

The primary outcome measure for this study was the presence of a Read (CTV3) code for an ASD recorded in a child’s primary care records. In collaboration with our primary care electronic health provider across Bradford, linkage was conducted using a complete deterministic match on National Health Service (NHS) number, surname, sex and date of birth (99% of BiB children were matched to their health record). Read codes are the standard clinical terminology system used in General Practice in the United Kingdom. A validated READ code list, based on Clinical Terms Version 3 [23] (Supplemental Table S1) has been shown in earlier published work [24] to be reliable and used with confidence to study ASD. Children were aged between 8 years 3 months and 12 years 6 months at the point of the primary care data extract (4th October 2019). Additional details of the data-linkage procedure can be found in the supplementary material.

### Statistical analysis

In order to account for variation in urine dilution during pregnancy [5], the standardised measure of urinary iodine to creatinine ratio (I:Cr) (μg/g) was used as the primary exposure, though all analyses were also conducted using UIC, including pre-specified sensitivity and subgroup analyses. To avoid categorising iodine concentrations, restricted cubic splines were fitted with four knots placed at percentiles 5, 35, 65 and 95 [25] and were used in all logistic regression models.

Multiple imputation by chained equations, based on 100 imputed datasets, was used to correct for the effects of incomplete covariate information [26] (details in Supplemental Table S2). Adjustment for confounding was informed by a directed acyclic graph (Supplemental Fig. S1). All reported results are from fully adjusted models controlling for maternal age (at study recruitment), ethnicity, socioeconomic and education category (see details in Supplemental Table S3) smoking in pregnancy, alcohol in pregnancy, pre-pregnancy body mass index (BMI) and parity as potential confounders. Covariates were omitted, as appropriate, for modelling different outcomes (see Supplemental Table S4).

Pre-specified sensitivity analyses were performed to assess the robustness of results which included: (i) excluding extreme iodine concentrations (outside 3 standard deviations (SD) of the mean on the log scale) (*n* = 41), (ii) the exclusion of iodine-containing supplement users (*n* = 1036) and (iii) excluding women with complications in pregnancy such as hypertension, gestational diabetes or preeclampsia (*n* = 909). Evidence of effect modification by child sex, ethnicity (White or Pakistani ethnic background), maternal socioeconomic and education category (‘more deprived and less educated’ or ‘less deprived and more educated’ (details in Supplementary Material, Table S3) were formally tested by including an interaction term in the model. Additional sensitivity analyses were completed using dietary data available in a sub-sample of the cohort (see Supplemental Table S4), for paternal age (years) and for seasonality, respectively, using pairs of sine and cosine functions which were prepared for each date across the year.

Logistic regression models were implemented using Stata version 15.1 (StataCorp) and a final parsimonious model was developed to determine the association between I:Cr and the probability of having an ASD diagnosis in the primary care records. The Wald test was used to assess the overall contribution of iodine status to each model. A two-tailed *p*-value < 0·05 was considered statistically significant and 95% confidence intervals (CIs) were used for all comparisons.

Figures present adjusted predicted outcomes (continuous variables) or probabilities (binary outcomes), across the range of iodine concentrations for an “average” participant, i.e. primiparous, white European, non-smoker, who did not report consuming alcohol, were employed and not materially deprived, of mean age (27·2 years) and BMI (25·8 kg/m^2^). The predicted probability of having a recorded diagnosis of ASD at the 25th and 75th percentiles of exposure were derived from these figures and tabulated (Table S5).

## Results

### Participant characteristics

Of 12,453 women with 13,776 pregnancies recruited into the cohort, 6644 women (53%) provided a total of 6977 urine samples at around 26–28 weeks’ gestation. In total, 6955 samples were successfully analysed for both iodine and creatinine from 6639 women who had a total of 7013 children. Women who provided urine samples were broadly similar to those in the cohort that did not (Supplemental Table S6). The mean age of participants was 27·2 years (SD 5·6) and mean pre-pregnancy BMI was 25.9 kg/m^2^ (SD 5·5) (Table 1). Descriptive maternal details and ASD diagnosis are presented by category of maternal urinary I:Cr in Tables 1 and 2, respectively.

**Table 1:** Maternal characteristics according to urinary iodine to creatinine ratio

		Iodine-to-creatinine ratio (cohort split into thirds)		
	All participants	lower third	middle third	higher third
I:Cr (μg/g), median (range)	83	59·5 (< 67)	83·1 (67 to 105)	121·9 (> 105)
N	6973	2325	2324	2324
I:Cr (μg/g), geometric mean (95% CI)	86·0 (85·0 to 87·2)	48·6 (48·1 to 49·2)	83·4 (82·9 to 83·8)	154·9 (154·9 to 159·3)
I:Cr (μg/g), median (IQR)	83·1 (59·3 to 121·0)	51·5 (42·8 to 59·5)	83·1 (74·4 to 93·3)	145·7 (121·0 to 185·9)
UIC (μg/L), geometric mean (95% CI)	70·8 (69·6 to 72·1)	45·9 (44·6 to 47·2)	69·9 (68·1 to 71·8)	110·6 (107·7 to 113·7)
UIC (μg/L), median (IQR)	76·3 (44·7 to 120·2)	52·1 (29·0 to 78·1)	77·4 (47·4 to 111·4)	117·8 (72·0 to 178·9)
Age (years), mean (SD)	27·2 (5·6)	26·5 (5·5)	27·3 (5·6)	27·9 (5·6)
BMI (kg/M^2^), mean (SD)	25·9 (5·5)	26·5 (5·7)	25·9 (5·4)	25·2 (5·1)
Socio-economic status^†^				
Least deprived and most educated	1313 (21)	323 (16)	456 (22)	534 (26)
Employed, not materially deprived	1299 (21)	353 (17)	458 (22)	488 (23)
Employed, no access to money	966 (16)	338 (16)	313 (15)	315 (15)
Receives benefits, not materially deprived	1671 (27)	666 (32)	527 (26)	478 (23)
Most economically deprived	954 (15)	378 (19)	304 (15)	272 (13)
Ethnic background, n (%)				
White British & European	3020 (43)	847 (36)	1061 (46)	1112 (48)
Pakistani	2951 (43)	1112 (48)	951 (41)	888 (38)
Other (Black, Indian, mixed, other)	964 (14)	353 (15)	299 (13)	312 (14)
Parity				
0	3196 (46)	1015 (44)	1055 (46)	1126 (49)
1	1896 (27)	617 (27)	640 (27)	639 (27)
2	1075 (15)	376 (16)	363 (16)	336 (15)
3+	806 (12)	317 (14)	266 (11)	223 (10)
Health and lifestyle in pregnancy				
Gestational diabetes, n (%)	525 (8)	165 (7)	174 (8)	186 (8)
Pre-pregnancy hypertension, n (%)	50 (1)	21 (1)	17 (1)	12 (1)
Pregnancy-induced hypertension, n (%)	383 (5)	128 (5)	127 (5)	128 (6)
Preeclampsia, n (%)	181 (3)	64 (3)	56 (2)	61 (3)
Drank any alcohol, n (%)	1323 (19)	386 (17)	476 (20)	461 (20)
Smoked, n (%)	1014 (15)	321 (14)	366 (16)	327 (14)
Iodine-containing supplements, n (%)	1036 (15)	134 (6)	279 (12)	623 (27)
Total fish intake^a^ (g/d), mean (SD)	23·8 (29·3)	20·9 (28·3)	25·3 (30·8)	25·3 (28·4)
Eat 5 fruit/vegetables per day^a^, n (%)				
Always	441 (18)	128 (15)	164 (20)	149 (19)
Sometimes	1782 (75)	628 (77)	595 (73)	559 (75)
Never	154 (6)	59 (7)	52 (6)	43 (6)

Abbreviations: *BMI* Body mass index, *CI* Confidence intervals, *I:Cr* Urinary iodine to creatinine ratio, *IQR* Interquartile range, *SD* Standard deviation, *UIC* Urinary iodine concentration

^a^Data from women who were asked about diet in pregnancy (*n* = 2202)

^†^See definitions in Supplemental Table S3

**Table 2:** Observed number of cases of autism spectrum disorder (ASD) both for the cohort as a whole (*n* = 92) and according to maternal iodine-to-creatinine ratio (I:Cr). In addition to the modelled predicted probability of a diagnosis of ASD (95% CIs) at the 25th, 50th and 75th centiles of I:Cr, for ‘average’ participants^a^

Iodine-to-creatinine ratio I:Cr (μg/g), median (range)	(n)	Observed cases of autism spectrum disorder (ASD) (n) (percent)	Probability of a diagnosis of autism spectrum disorder (percent) (95%CIs)
All participants:	6973	92 (1·3%)	
Lower third: 59·5, (0·7 to 67)	2325	28 (1·2%)	1·0 (0·3 to 1·7)
Middle third: 83·1, (67 to 105)	2324	27 (1·2%)	1·1 (0·4 to 1·8)
Higher third: 121·9, (> 105)	2324	37 (1·6%)	1·5 (0·5 to 2·4)

^a^ ‘Average’ participants were specified according to the largest categories or mean cohort values. Predicted estimates represent an average birth, not specified by child’s sex - related to participants who are primiparous, white ethnic background, ‘Employed and not materially deprived’, did not smoke, drink, were of mean age and an average BMI

The median (inter-quartile range (IQR)) UIC for all samples was 76 μg/L (45 to 120) with 29% below 50 μg/L (Table 1), indicating a deficient population according to WHO guidelines [20]. In White European women, the median UIC (82 μg/L, IQR 49 to 127) was higher than women of Pakistani (73 μg/L, IQR 42 to 115) or other ethnic background (72 μg/L, IQR 42 to 111) (Supplementary Material, Table S7). Median (IQR) I:Cr was 83 μg/g (59 to 121) and when the cohort was divided into five groups according to I:Cr, women with lower I:Cr were more likely to be grouped in the lower socioeconomic and education categories (Table 1).

### Diagnosis of an ASD

Currently, around 1% of children and adults in the UK have a recorded diagnosis of ASD [27]. In our cohort, 92 children (1·3%) had received a diagnosis of ASD by the census date. Boys had a higher rate of recorded ASD diagnosis than girls, with approximately 2·0% of boys having a Read code indicating a diagnosis of ASD in their primary care records compared to 0·6% of girls (Supplemental Table S8).

**Fig. 1:**
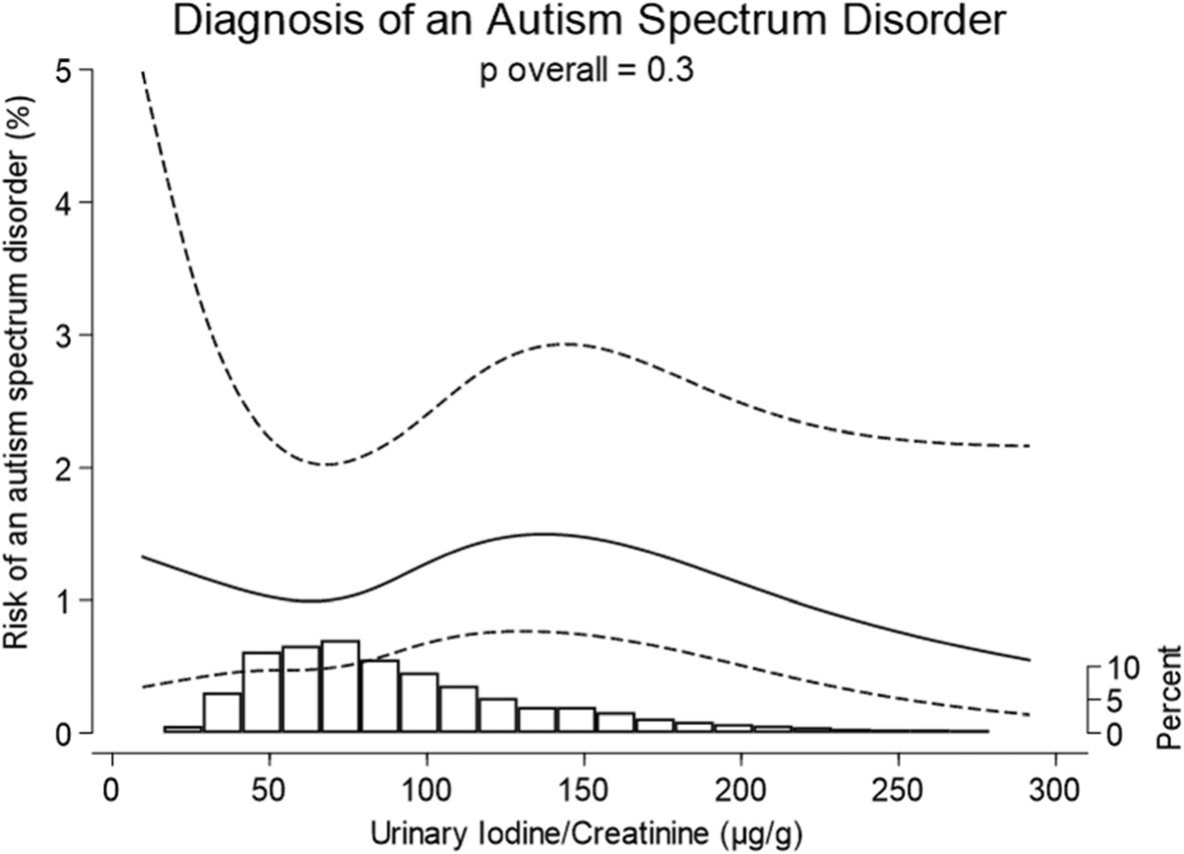
Estimated risk of an autism spectrum disorder for children (aged 8 to 12 years) of typical mothers, across a range of maternal I:Cr concentrations (μg/g). Histograms illustrate the distribution of iodine concentrations and although the figures are curtailed at 300 μg/g, the splines (solid line) and 95% CIs (dashed lines) were drawn using data from all participants. Splines were drawn after adjustment for confounders (details in Supplemental Table S4). CIs are particularly wide for very low values of Urinary Iodine/Creatinine. This reflects the very small numbers of women in our sample with extremely low intakes of iodine (only 3·7% of participants have an I:Cr < 35 μg/g) and does not rule out the potential for a more visible trend in more severely deficient populations. The spline position in these figures illustrates the predicted estimate for typical participants (primiparous, white ethnic background, ‘Employed and not materially deprived’, did not smoke, drink or experience complications in pregnancy, of mean age and average BMI

There was no evidence of any association between I:Cr and the risk of a diagnosis of ASD after adjusting for established confounders (p_overall_ 0·3) (Fig. 1). Figures present the adjusted predicted probability of the diagnosis of an ASD, across the range of iodine concentrations for an “average” participant, as previously described. For a mother with typical characteristics (defined above) the probability of an ASD diagnosis at the 25th percentile of I:Cr (59 μg/g) was estimated to be 1·0% (0·3–1·7) and at the 75th percentile of I:Cr (121 μg/g) was estimated as 1.5% (0·5–2·4), respectively (difference = 0·5 percentage points (95%CI: − 0·1–1·1, *p* = 0·1). The trend remained broadly flat across the observed range of I:Cr values. All results were broadly similar using UIC (Supplementary Material, Fig. S2).

### Subgroup and sensitivity analysis

In subgroup analysis, the association between I:Cr and autism was similar in boys (*p* = 0.2) and girls (*p* = 0.1) (p interaction = 0·05). There was no evidence that the association between I:Cr and the probability of having an ASD diagnosis in the primary care records was different according to ethnic background (p_overall_ = 1.0) or maternal deprivation (p_overall_ = 0·3). Estimates from a sensitivity analysis using only women with complete data were not substantively different from primary analyses using imputed data (*p* = 0.3) and were also similar after excluding iodine-containing supplement users (*p* = 0.5) and extreme I:Cr values (*p* = 0.3) (see Supplemental Fig. S3 a-d). There was no evidence of any association in the full sample or of differences between subgroups, across maternal I:Cr (Supplemental Fig. S3 e-j) and UIC (Supplement Fig. S4 a-f).

## Discussion

The present study found no evidence to support a link between insufficient iodine status in the second-third trimester and an increased risk of ASD in the child, in a large multi-ethnic cohort with mild-to-moderate iodine deficiency, in a setting where there was no routine dietary fortification or supplementation. Children born to mothers with lower iodine status were of no greater probability of having a clinical diagnosis of ASD in their primary care records compared to those born to mothers with higher iodine status.

We found no evidence to indicate any optimum iodine status or any threshold associated with a heightened risk of ASD. However, confidence intervals were wide, especially at the lower range of exposure reflecting the small proportion of participants with I:Cr measurements at very low levels. Therefore, we cannot exclude the possibility of iodine deficiency being associated with autism, but the study was underpowered to detect small differences in risk.

A small number of studies have reported associations between inadequate thyroid hormone supply during the first trimester of pregnancy and increased ASD risk in children [14–16], yet the role of iodine in these associations remained uncertain. Despite the mechanistic link between iodine and thyroid hormone synthesis, this is to our knowledge only the second prospective study to date to investigate any direct link between maternal iodine status and ASD [17].

Findings from the present study are consistent with a recent cohort pooling project, combining data from 3 large population-based cohorts [17], which also found no consistent evidence to suggest that a lower maternal iodine status (< 150 μg/g) at < 14 weeks nor < 18 weeks gestation was associated with higher parent-reported autistic trait score in children. Whilst this study used an objective biomarker to measure maternal iodine status, the ascertainment of autistic traits was performed at different ages by different instruments and evaluators, across the 3 cohorts. This may have potentially introduced some heterogeneity. Furthermore, this supporting study [17] lacked medical-record data to confirm ASD diagnosis. A major strength of our current study lies in our consistent methodology and capture of clinical outcome through data linkage with primary care records of the cohort children. This reduces scope for subjectivity in the outcome assessment and thus, minimises bias from misclassification. The present study remains unique in the objective measure used to inform our ASD clinical outcome.

Iodine deficiency remains one of the leading causes of hypothyroidism, both overt and subclinical [28]. However, hypothyroidism during pregnancy can also be a secondary consequence of a number of conditions such as inflammatory disease and autoimmune disorders [29], not just iodine deficiency. Likewise, thyroid hormones can also be altered by air pollution, pesticides, and exposure to persistent organic pollutants [30], pollutants which have also been suggested to increase the risk of ASD in children [31]. Therefore, when relying on measures such as maternal thyroid parameters and recorded diagnoses of pre and perinatal overt hypothyroidism [14, 15] it is not possible to determine whether these observed associations with ASD are directly related to iodine status or confounded by other environmental exposures. A key strength of our study is the use of a valid biomarker of iodine status in pregnant women (I:Cr) [32] which accounts for urine dilution variability and, when used in conjunction with our population approach, permits direct and novel exploration of a spectrum of iodine exposure on clinical ASD outcome, without needing to rely on intermediate thyroid hormone measures.

Estimating iodine status from single spot urine samples may introduce potential exposure measurement error because of day-to-day variation in iodine intake and excretion that do not reflect true long-term iodine status, so attenuating our results. However, intra-individual variation in urine volume was reduced by correcting for urinary creatinine concentration. In particular, this has been shown to reduce variation, and provide stronger correlation with 24 h urines, in same-sex groups of a similar age, such as our cohort of young women [33, 34]. Furthermore, using 24 h urines may have resulted in lower participation rates, potentially reducing both power and generalisablity.

Timing of urine samples could potentially account for differences between studies. Peak neuronal migration occurs in humans between the 12th and 20th weeks of gestation [35], resulting in the formation of the cortical plate. If iodine insufficiency during a vulnerable window prior to 26 weeks gestation were to impair neuronal migration and if this increased risk of ASD by that mechanism [36], then our findings may not fully reflect this process. However, some studies have failed to find associations between iodine and ASD at earlier timepoints [8], whilst others have found associations between neurodevelopmental outcomes and iodine measured both prior to conception [37] and at later time points [17]. It is therefore not known if other vulnerable periods exist prior to conception or during early life [37] and other mechanisms could exist including later neuronal migration [38]. UIC at 26 weeks will correlate with iodine status at both earlier and later time points, so our findings remain of relevance, but there is scope for additional investigation relating to timing of exposure.

It is possible that results from our study, or previous studies, may in part reflect residual confounding not accounted for in regression models. However, we considered all established risk factors within our causal framework and adjusted for those identified as confounders. Other pre-natal factors such as parental psychiatric history and medication use, especially in relation to maternal mental health, all have reported affiliations with ASD [31] and warrant further consideration.

ASD has low prevalence in the community [27], so the numbers of cases were relatively low. We were therefore unable to explore some subgroups of interest. It remains possible that potential cases have been missed if not diagnosed by the census date. It is possible that such cases could differ in presentation or cause from those identified in this current work. However, the long follow-up to age 12 years and complete record linkage has likely identified most cases, including those where diagnosis has been delayed.

## Conclusions

The present study found no evidence to support an association between mild-moderate iodine deficiency at the end of the second trimester and a subsequent clinical diagnosis of ASD in the offspring by age 12. Low incidence of the outcome in the study population leads to low power to detect weaker associations. As such, this study does not rule out the potential for some impact of low maternal iodide intake on ASD or other outcomes in other populations or in future studies with 24-h urines collected through pregnancy, in particular in trimester 1, which would improve precision and target potentially earlier critical windows of exposure.

## Supplementary Information

**Additional file 1.**

## Data Availability

The data for this the current study was used under a limited use data use agreement between The Bradford Institute for Health Research and the University of Leeds. The data that support the findings of this study are available from the University of Leeds; but restrictions apply to the availability of these data because of the need to maintain participant confidentiality. The datasets used and/or analysed during the current study are available from the corresponding author on reasonable request.

## Abbreviations

ASD: Autism Spectrum Disorder
UIC: Urinary Iodine Concentration
I:Cr: Iodine Creatinine Ratio
BiB: Born in Bradford
WHO: World Health Organization
ADHD: Attention Deficit Hyperactivity Disorder
CDC: Centers for Disease Control and Prevention
EQUIP: Ensuring the Quality of Urinary Iodine Procedures
QC: Quality Control
CRM: Certified Reference Material
NHS: National Health Service
GP: General Practice
BMI: Body Mass Index
CI: Confidence Interval
SD: Standard Deviation
IQR: Inter-Quartile Range
