# Supplementary material for: Maternal iodine status in a multi-ethnic UK birth cohort: associations with autism spectrum disorder
Source: BMC Pediatr. 2020 Dec 5;20:544. doi: 10.1186/s12887-020-02440-y (PMC7718710; doi:10.1186/s12887-020-02440-y)
Supplement: Supplementary file 1 — Additional file 1. [file 12887_2020_2440_MOESM1_ESM.docx]

**Supplementary material**

**Maternal iodine status in a multi-ethnic UK birth cohort: associations with autism spectrum disorder.**

**Additional laboratory analysis details**

Sample concentration was determined against a urine matrix matched standard curve spiked with 0, 5, 10, 40, 70, 100, 400 and 800µg/L iodide (Sigma Aldrich).

A series of quality control (QC) urines with previously determined UIC ranges were analysed alongside participant samples. The first series consisted of QC urine A (target iodine value: 71·6µg/L, 60.2 – 80.1), B (target iodine value: 103·6µg/L, 87·5 – 118·9) and C (target iodine value: 164·1µg/L, 133·4 – 189·8). The second series replaced QC urine A with QC urine D (target iodine value: 56·8µg/L, 44·8 – 66·4) due to QC urine depletion. The third series replaced QC urine C with QC urine E (target iodine value: 155·6µg/L, 139·7 – 173·4), again due to depletion. Target urine values and ranges were determined via the three step procedure outlined in the Centers for Disease Control and Prevention (CDC)^1^ EQUIP standardisation programme. Participant samples, quality control samples, internal standards and certified reference materials were vortexed, centrifuged, and diluted 1:10 before analysis. Final samples consisted of 500 µL of urine, 4000µL of diluent (1% tetramethylammonium hydroxide (Sigma Aldrich), 0.01% Triton X-100 (Sigma Aldrich)). Diluent was spiked with 10 µg/L of tellurium to act as an internal ICP-MS reference standard. A certified reference material (Seronorm Trace Metal Urine Level 1) was included in each run to provide external validation (target iodine value 105µg/L, certified range: 84-126).

Observed mean values for QC urines across the course of the study were 70·7μg/L (n=99), 103·2μg/L (n=410) and 161·6μg/L (n=150) for urines A, B and C respectively. The observed values for QC urines D and E were 55.6μg/L (n=329) and 156.5μg/L (n=261). Certified reference material gave a mean value of 107.7μg/L across all runs.

Assessment of intra-run precision for QC samples gave coefficients of variation (CV) of 1.03% at 71.5μg/L (A), 2.43% at 101.3μg/L (B), 1.57% at 160.1μg/L (C), 2.67% at 58.0μg/L (D) and 1.06% at 145.0μg/L (E). Inter-run precision measures for QC samples gave a CV of 7.5% at 70.7μg/L (A), 7.6% at 103.2μg/L (B), 8.7% at 161.6μg/L (C), 9.7% at 55.6μg/L (D) and 5.4% at 156.5μg/L (E). Assessment of intra-run precision for certified reference material gave a CV of 1.76% at 106.4μg/L. Assessment of inter-run precision for certified reference material gave a CV of 5.5% at 107.7μg/L.

The method limit of quantification was 1.46μg/L.

Urinary creatinine concentrations were assessed through a standard microplate assay utilising the Jaffe reaction. Assessment of creatinine intra-run precision gave a CV of 2% at 10 mg/L, 1% at 70mg/L and 1.1% at 120mg/L. Assessment of inter-run precision gave a CV of 14.5% at 10mg/L, 9.7% at 70mg/L and 5.5% at 120mg/L.

**Subgroup and sensitivity analysis**

Sensitivity analyses were conducted to assess the robustness of results: (i) excluding extreme iodine concentrations (i.e. outside 3 standard deviations (SD) of the mean on the log scale) (n=41), (ii) excluding women with complications in pregnancy (gestational diabetes, hypertension or preeclampsia) (n=906), and (iii) to exclude users of iodine-containing supplements (n=1036). Additional sensitivity analyses were completed using dietary data available in a sub-sample of the cohort (Table S4), for paternal age (years) and for seasonality, respectively, using pairs of sine and cosine functions which were prepared for each date across the year.

Models were also run separately by ethnic group (White or Pakistani ethnic background) and by maternal socioeconomic and education category ('more deprived and less educated' or 'less deprived and more educated' (Table S3) and by child sex (male or female). Though socioeconomic and education category was included in its five original categories for main analysis, for the purpose of formally testing subgroup interaction, two categories were used.

**Data-linkage procedure**^3^

Linkage was conducted, in partnership with our primary care electronic health provider across Bradford, using a complete deterministic match on National Health Service (NHS) number, surname, gender and date of birth. Routine electronic datasets comprise primary care data from the GP practice, hospital data, community care and education data. Hospital and maternity data were provided by Bradford Teaching Hospitals NHS Foundation Trust (BTHFT). The community care data were provided by Bradford District Care Trust. In collaboration with our local provider of electronic health records across primary care, the GP records were extracted by The Phoenix Partnership, SystmOne.

**Table S1:** Read (CVT3) codes for autism

| **Read (CVT3) code** | **Read (CVT3) code description** |
| --- | --- |
| X00TM | Autism spectrum disorder |
| XE2v2 | Childhood autism |
| XabEY | Under care of autism assessment service |
| X00TN | Atypical autism |
| E1400 | Active infantile autism |

Read codes identified for 92 children with at least one read (CTV3) code for autism in their primary care records.

**Multiple imputation details**

Multiple imputation by chained equations, based on 100 imputed datasets, was used to correct for the effects of incomplete covariate information.^2^ All model covariates were used in imputation, including the outcome and iodine splines. Imputation models were used for analyses using I:Cr or UIC. Predictive mean matching was used for continuous variables and logistic regression for categorical, with 100 imputed estimates.

**Table S2:** Missing data imputed

| Variable | Missing data out of 6973 pregnancies* |
| --- | --- |
| BMI | 866 |
| Ethnicity | 38 |
| Socioeconomic & education category | 770 |
| Smoking status | 754 |
| Alcohol use in pregnancy | 789 |
| Length of Gestation | 179 |

*Note that missing data numbers varied for each imputation model according to the presence of relevant outcomes. These numbers relate to the whole sample.

**Table S3:** Details of socioeconomic position categories and maternal education levels

| N (%) | | **Less deprived and more educated**  (derived using three categories from latent class analysis*   1. Least socioeconomically deprived and most educated (n=1313), 2. Employed and not materially deprived (n=1299), 3. Employed and no access to money (n=966)) | **More deprived and less educated**  (derived using two categories from latent class analyses*   1. Receiving benefits and not materially deprived (n=1671), 2. Most economically deprived (n=954)) |
| --- | --- | --- | --- |
| Highest maternal educational achievement | |  |  |
|  | <5 GCSE equivalent | 145 (4%) | 1125 (43%) |
|  | 5 GCSE equivalent | 843 (24%) | 975 (37%) |
|  | A-level equivalent | 733 (20%) | 199 (8%) |
|  | Higher than A-level | 1527 (43%) | 170 (6%) |
|  | Don't know/ other | 330 (9%) | 156 (6%) |
|  |  |  |  |
| In receipt of means-tested benefits | | 657 (18%) | 1740 (66%) |

*The 5 original socioeconomic position categories were derived from latent class analysis, to group women with similar socioeconomic position profiles.^4^ To do this, 19 determinants were included in the analysis, including maternal and paternal employment, maternal and paternal education, receipt of benefits, housing tenure and the ability to save or buy goods and services. These 5 categories were then divided into two groups to approximately represent women who were 'Less deprived and more educated' or 'More deprived and less educated'.

**Table S4:** Details of model covariates, sample exclusions, sensitivity analyses and subgroup analyses for the risk of an autism spectrum disorder. All analyses ran with both I:Cr (µ/g) and UIC (µ/L).

|  |  | |  |
| --- | --- | --- | --- |
| M0 | **M2** | **M3** | **Model covariates** |
|  | ✓ | ✓ | Maternal age (years) at recruitment (26-28 weeks gestation) |
|  | ✓ | ✓ | Socioeconomic and education category^1^ |
|  | ✓ | ✓ | Ethnicity (White, Pakistani, Other) |
|  | ✓ | ✓ | BMI (pre-pregnancy), Kg/m^2^ |
|  | ✓ | ✓ | Parity (0, 1, 2, 3+) |
|  | ✓ | ✓ | Smoked in pregnancy (yes/no) |
|  | ✓ | ✓ | Alcohol in pregnancy (yes/no) |
|  |  | ✓ | Eats 5 fruit/vegetables per day (Never, sometimes, always) |
|  |  | ✓ | Total fish intake (g/week) |
|  |  | ✓ | Season of urine sample collection |
|  |  | ✓ | Paternal age (years) |
|  |  |  | **Sensitivity analysis** |
|  |  |  | Exclude extreme iodine values |
|  |  |  | Exclude iodine-containing supplement users |
|  |  |  | Exclude pregnancy complications (diabetes, hypertension, pre-eclampsia) |
|  |  |  | **Subgroup analysis** |
|  |  |  | Ethnic group (White & Pakistani origin) |
|  |  |  | Socioeconomic and education position in 2 categories^2^ |
|  |  |  | Child sex (Male vs Female) |

^1.^ Five categories of socioeconomic and education position (see Table S3 for further details)

^2.^ Two categories of socioeconomic and education position (see Table S3 for further details)

M0: Unadjusted model

**M2:** Main model including confounders identified using directed acyclic graph (DAG) (Figure S1). These results are reported in manuscript.

Sensitivity analysis in cohort subgroup with details of paternal age (n=1942) and dietary data available (n=2202) in a subsample of the cohort. Additional adjustments included total fish intake (g/day) and consuming five portions of fruit and vegetables per day (never/ sometimes/ always) and seasonality.

Sensitivity analysis was performed to observe the effect of removing child sex from the main model (M2). Estimates from the altered model with child sex removed (p=0.3) were not materially different from the those derived from the main model used for analysis which included adjustment for child sex.

**Table S5.** Predicted percent at the threshold (95% CIs) at the 25^th^, 50^th^ and 75^th^ centiles of iodine concentration (I:Cr and (UIC) and p-_overall_* for ‘average’ participants

|  |  | **Iodine to creatinine ratio (µg/g)** | | | | | **Urinary iodine concentration (µg/L)** | | | | |
| --- | --- | --- | --- | --- | --- | --- | --- | --- | --- | --- | --- |
|  |  | **25^th^ centile**  59µg/g | **50^th^ centile**  83µg/g | **75^th^ centile**  121µg/g | **25^th^ vs. 75^th^ centile** difference | P _overall_^‡^ | **25^th^ centile**  45µg/L | **50^th^ centile**  76µg/L | **75^th^ centile**  120µg/L | **25^th^ vs. 75^th^**  centile difference | P _overall_^‡^ |
| Probability of a diagnosis of autism spectrum disorder. | Percent  (95% CIs) | 1.0  (0.3 to 1.7) | 1.1  (0.4 to 1.8) | 1.5  (0.5 to 2.4) | 0.5  (-0.1 to 1.1) | 0.3 | 1.2  (0.4 to 2.0) | 1.2  (0.4 to 2.0) | 1.2  (0.3 to 2.1) | 0.0  (-0.6 to 0.5) | 0.9 |

*p-_overall_ is derived from comparing restricted cubic spline models with and without iodine status included. p<0·01 indicates a significant association between iodine and autism spectrum disorder and the association may be linear or non-linear in nature.

† 'Average' participants were specified according to the largest categories or mean cohort values. Predicted estimates represent an average birth, not specified by child's sex - related to participants who are primiparous, white ethnic background, 'Employed and not materially deprived', did not smoke, drink, are of average age and have an average BMI.

‡ Note that p-values are unaffected by specifying 'average participants' and therefore relate to observed associations in the whole cohort.

**Table S6:** Characteristics of mothers with usable urine samples and the rest of cohort

|  | | **Participants that provided urine sample** (n) (%) | **Rest of cohort**  (n) (%) |
| --- | --- | --- | --- |
| N (% of total cohort) | | 7060 (51) | 6897 (49) |
| Age (years), mean (SD) | | 27·2 (5·6) | 27·0 (5·6) |
| BMI (Kg/M^2^), mean (SD) | | 25·9 (5·4) | 26·4 (5·9) |
| Educational achievement^1^, n (%) | |  |  |
|  | <5 GSCE equivalent | 1291 (18) | 1200 (17) |
|  | 5 GSCE equivalent | 1849 (26) | 1681 (24) |
|  | A-level equivalent | 945(13) | 725 (11) |
|  | Higher than A-level | 1726 (24) | 1220 (18) |
|  | Don’t know/ other | 1249 (18) | 2071 (30) |
| Socio-economic status | |  |  |
|  | Least deprived and most educated | 1328 (21) | 932 (18) |
|  | Employed and not materially deprived | 1311 (21) | 970 (19) |
|  | Employed and no access to money | 974 (16) | 775 (15) |
|  | Receiving benefits and not materially deprived | 1697 (27) | 1674 (32) |
|  | Most economically deprived | 963 (15) | 859 (16) |
| Ethnic background, n (%) | |  |  |
|  | White European | 3044 (43) | 2697 (40) |
|  | Pakistani | 2988 (43) | 3263 (48) |
|  | Other (Black, Indian, mixed, other) | 981 (14) | 859 (13) |
| Health and lifestyle in pregnancy | |  |  |
|  | Complication in pregnancy^3^, n (%) | 912 (13) | 882 (13) |
|  | Drank any alcohol, n (%) | 1334 (19) | 932 (14) |
|  | Smoked, n (%) | 1024 (15) | 872 (13) |
|  | Used supplements, n (%) | 1409 (20) | 877 (13) |
|  | Used iodine-containing supplement, n (%) | 1048 (15) | 649 (9) |
|  | White fish intake^2^ (g/d), mean (SD) | 20·9 (26·9) | 21·6 (29·5) |
|  | Oily fish intake^2^ (g/d), mean (SD) | 1·4 (3·9) | 1·3 (4·2) |
|  | Total fish intake^2^ (g/d), mean (SD) | 23·9 (29·4) | 24·4 (33·4) |
|  |  |  |  |

Note, n relates to pregnancies registered within BiB and thus, includes mother data twice for those who took part in the study with multiple pregnancies.

Abbreviations: BMI body mass index; CI confidence intervals; GCSE General Certificate of Secondary education; IQR interquartile range; SD standard deviation.

^1^UK-equivalent qualifications were calculated from detailed overseas qualifications. GCSEs are standard assessments at age approx. 16. A-level assessments indicate education to approx. age 18.

^2^ Mean calculated from among those who were asked about diet in pregnancy (n=2202)

^3^ Gestational diabetes, hypertension or pre-eclampsia

**Table S7:** Maternal characteristics according to ethnic group, n= 6973 mothers

|  | |  | **Ethnic background** | | |
| --- | --- | --- | --- | --- | --- |
|  | | All participants | White European | Pakistani | Other |
|  | | n=6973 | n=3020 | n=2951 | n=964 |
| I:Cr (µg/g), geometric mean (95% CI) | | 86.0 (85.0 to 87.2) | 91·2 (89.5 to 93·0) | 81·6 (80.1 to 83·3) | 84.4 (81·3 to 87.5) |
| I:Cr (µg/g), median (IQR) | | 83.1 (59.3 to 121.0) | 88·4 (64·0 to 128·9) | 79.0 (56·2 to 114·4) | 80·3 (55·5 to 119.5) |
| UIC (µg/L), geometric mean (95% CI) | | 70.8 (69.6 to 72.1) | 75·7 (73·7 to 77·7) | 67·4 (65·6 to 69.4) | 66·3 (63·1 to 69.7) |
| UIC (µg/L), median (IQR) | | 76.3 (44.7 to 120.2) | 81·6 (48·5 to 127·0) | 72·5 (41·9 to 114·3) | 72·8 (41·2 to 111·4) |
| UCC (mg/L), median (IQR) | | 0·90 (0·54 to 1·34) | 0·91 (0·56 to 1·32) | 0·90 (0·54 to 1·37) | 0·87 (0·51 to 1·34) |
| Maternal age (years), mean (SD) | | 27·2 (5·6) | 26·8 (5·9) | 27·5 (5·2) | 28·0 (5·4) |
| Paternal age (years), mean (SD) | | 30.7 (6.5) | 30.2 (6.8) | 30.9 (6.2) | 32.2 (5.8) |
| BMI (Kg/M^2^), mean (SD) | | 25·9 (5·5) | 26·3 (5·7) | 25·4 (5·2) | 25·8 (5·4) |
| Educational achievement^1^, n (%) | |  |  |  |  |
|  | <5 GSCE equivalent | 1277 (18) | 495 (16) | 684 (23) | 98 (10) |
|  | 5 GSCE equivalent | 1823 (26) | 873 (29) | 795 (27) | 155 (16) |
|  | A-level equivalent | 937 (13) | 493 (16) | 342 (12) | 102 (10) |
|  | Higher than A-level | 1705 (24) | 659 (22) | 707 (24) | 339 (35) |
|  | Don’t know/ other | 1231 (18) | 500 (16) | 423 (14) | 270 (28) |
| Socio-economic status | |  |  |  |  |
|  | Least deprived and most educated | 1313 (21) | 542 (19) | 493 (19) | 278 (36) |
|  | Employed and not materially deprived | 1299 (21) | 933 (33) | 247 (9) | 119 (15) |
|  | Employed and no access to money | 966 (15) | 438 (16) | 401 (15) | 127 (16) |
|  | Receiving benefits and not materially deprived | 1671 (27) | 393 (14) | 1144 (44) | 134 (18) |
|  | Most economically deprived | 954 (15) | 501 (18) | 342 (13) | 111 (15) |
| Health and lifestyle in pregnancy | |  |  |  |  |
|  | Complication in pregnancy^3^, n (%) | 914 (13) | 341 (11) | 438 (15) | 120 (12) |
|  | Drank any alcohol, n (%) | 1323 (19) | 1213 (40) | 14 (0·5) | 96 (10) |
|  | Smoked, n (%) | 1014 (15) | 875 (29) | 81 (3) | 58 (6) |
|  | Used iodine-containing supplement, n (%) | 1036 (15) | 536 (18) | 349 (12) | 151 (16) |
|  | Total fish intake^2^ (g/d), mean (SD) | 23·8 (29·3) | 19·4 (23·0) | 27·1 (32·2) | 33·3 (39·4) |

Abbreviations: BMI body mass index; CI confidence intervals; GCSE General Certificate of Secondary education; I:Cr urinary iodine to creatinine ratio; IQR interquartile range; SD standard deviation; UCC urinary creatinine concentration; UIC urinary iodine concentration.

^1^UK-equivalent qualifications were calculated from detailed overseas qualifications. GCSEs are standard assessments at age approx. 16. A-level assessments indicate education to approx. age 18.

^2^ Mean calculated from among those who were asked about diet in pregnancy (n=2776)

^3^ Gestational diabetes, hypertension or pre-eclampsia

**Table S8.** Recorded number of cases of autism spectrum disorder for all participants in the cohort (n=) and according to sex of child, socio-economic status^†^ and ethnicity.

| Cohort split by | | (n) | **Observed cases of autism spectrum disorder (ASD)** (n) (%) |  |
| --- | --- | --- | --- | --- |
| All participants | | 6973 | 92 (1.9%) |  |
| **Sex of Child** | |  |  | p_overall_=0.05 |
|  | Male | 3577 | 73 (2.0%) |  |
|  | Female | 3396 | 19 (0.6%) |  |
| **Socio-economic Status^†^** | |  |  | p_overall_= 0.3 |
|  | Least deprived, most educated | 3578 | 56 (1.6%) |  |
|  | Most economically deprived, least educated | 2625 | 24 (0.9%) |  |
| **Ethnicity** | |  |  | p_overall_= 1.0 |
|  | White British | 3020 | 47 (1.6%) |  |
|  | Pakistani | 2951 | 32 (1.1%) |  |

^†^ Binary socioeconomic class and education category variable made from latent class analysis.

*p_overall_ = overall p value for each adjusted model with an interaction term to explore the effect modification by each respective variable in the association between I:Cr and the probability of an ASD diagnosis.

**Figure S1.** A directed acyclic graph (DAG) used to identify potential confounders in the association between maternal iodine concentration and autism spectrum disorder.


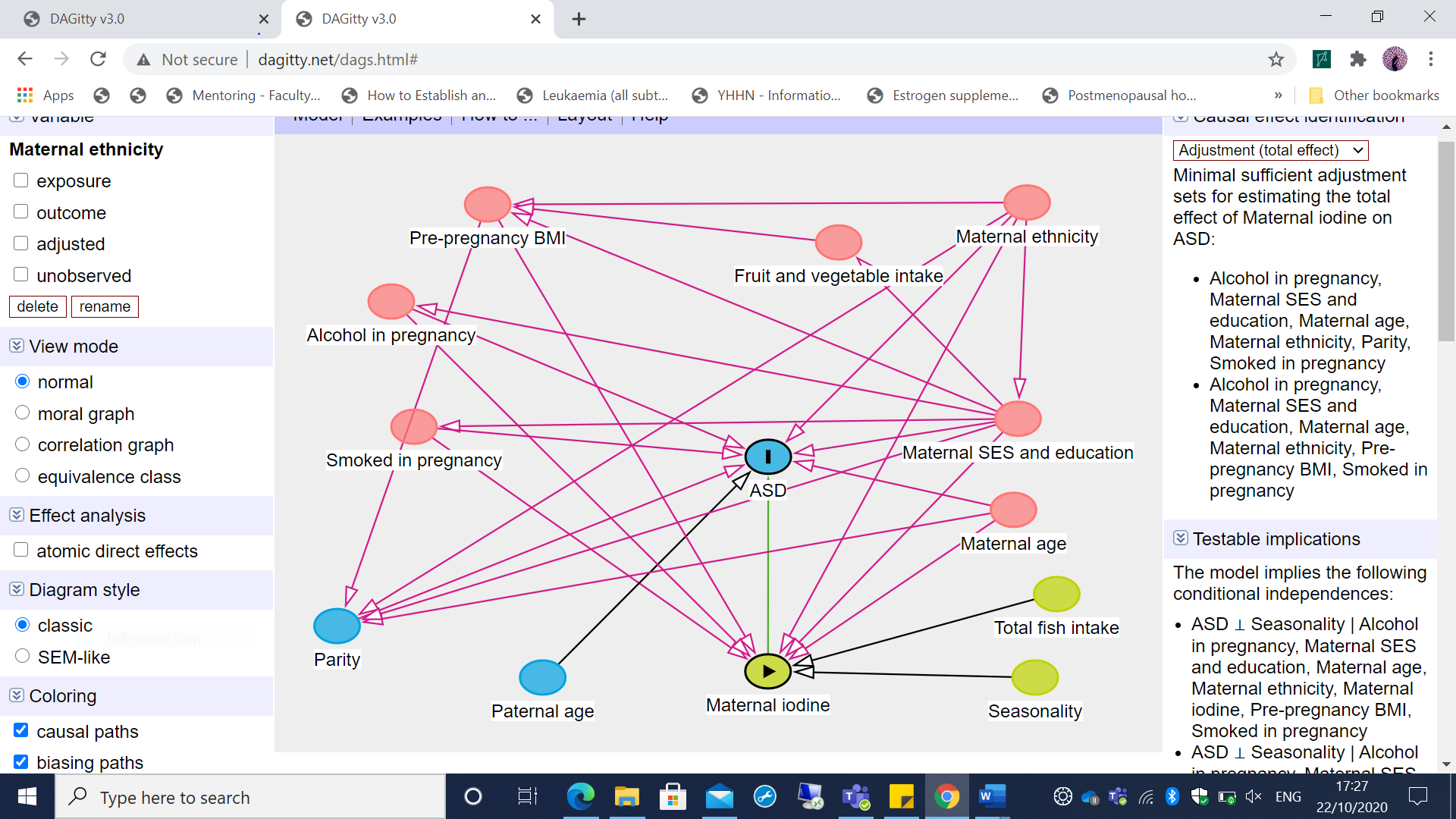


*Lines with arrows indicate potential causal associations.

Constructed using DAGitty software^5^.

**Figure S2.** Estimated risk of an autism spectrum disorder for children (aged 8 to 12 years) of typical mothers, across a range of maternal UIC concentrations (µg/L).

Histograms illustrate the distribution of urinary iodine concentrations and although the figures are curtailed at 300µg/g, the splines (solid line) and 95% CIs (dashed lines) were drawn using data from all participants. Splines were drawn after adjustment for confounders (Table S4).

The spline position in these figures illustrates the predicted estimate for typical participants (primiparous, white ethnic background, 'Employed and not materially deprived', did not smoke, drink or experience complications in pregnancy, are of average age and have an average BMI).

**Figure S3.** Probability of an autism spectrum disorder diagnosis in all participants (a), sensitivity analysis (b-d) and subgroups (e-h) across a range of maternal I:Cr concentrations (µg/g). With corresponding p-values from respective sensitivity/subgroup analysis.

**Pre-specified sensitivity**

**Figure S3 (e-j): subset analyses** – across maternal I:Cr (µg/g)

**Subgroup analyses** – across maternal UIC (µ/L)

**Figure S4 (a-f).** Probability of an autism spectrum disorder diagnosis in subgroups (a-f) across a range of maternal UIC concentrations (µg/L). With corresponding p-values from respective subgroup analysis.

**References**

1. Caldwell KL, Makhmudov A. The Challenge of Iodine Deficiency Disorder: A Decade of CDC’s Ensuring the Quality of Urinary Iodine Procedures Program: Centers for Disease Control and Prevention, 2011.
2. White IR, Royston P, Wood AM. Multiple imputation using chained equations: Issues and guidance for practice. *Statistics in medicine* 2011; **30**(4): 377-99.
3. Wright B, Mon-Williams M, Kelly B, Williams S, Sims D, Mushtaq F et al. Investigating the association between early years foundation stage profile scores and subsequent diagnosis of an autism spectrum disorder: a retrospective study of linked healthcare and education data. *BMJ Paediatrics Open*. 2019;3(1):e000483.
4. Fairley L, Cabieses B, Small N, et al. Using latent class analysis to develop a model of the relationship between socioeconomic position and ethnicity: cross-sectional analyses from a multi-ethnic birth cohort study. *BMC public health* 2014; **14**: 835.
5. Textor J, van der Zander B, Gilthorpe MS, Liskiewicz M, Ellison GT. Robust causal inference using directed acyclic graphs: the R package 'dagitty'. *International journal of epidemiology* 2016; **45**(6): 1887-94.
